# Supplementary material for: Dunaliella salina Alga Protects against Myocardial Ischemia/Reperfusion Injury by Attenuating TLR4 Signaling
Source: Int J Mol Sci. 2023 Feb 15;24(4):3871. doi: 10.3390/ijms24043871 (PMC9963554; doi:10.3390/ijms24043871)
Supplement: Supplementary file 1 [file ijms-24-03871-s001.zip › ijms-1884666-supplementary.pdf]

Supplementary material and methods

Cell culture

The H9c2 embryonic rat heart-derived (ventricular) cells (myoblasts) was purchased from AddexBio (cat no. C00031002) and cultured in Dulbecco's modified Eagle's medium (DMEM) supplemented with 10% fetal bovine serum (FBS), 1% penicillin (10,000 units/mL) and streptomycin (10,000 μg/mL) both from GIBCO (Grand Island, NY, USA). Cells were maintained in a humidified incubator consisting of 5% CO_2 and 95% air at 37°C.

Oxygen Glucose Deprivation/Reoxygenation (OGD/R) Model

In order to mimic an *in vitro* model of myocardial I/R injury, H9c2 cells were subjected with the oxygen and glucose deprivation followed by reoxygenation (OGD/R). H9c2 cells were seeded onto 12-well plates at a density of 1×10^4 per well for 24 hours. The medium was replaced and the cells were exposed to the different treatment: Control (0.1% DMSO) and *D. salina* (10 mM) for 1 hr before OGD. To simulate anoxia in H9c2 cells, plated cells were exposed to a glucose-free and serum-free solution and incubated for 16 hours in direct heat CO_2/multi-gas incubator (astec, japan) saturated with 95% N_2 and 5% CO_2 at 37°C. Then, the cells were incubated with normal culture medium in CO_2 incubator (NAPCO) saturated with 95% air and 5% CO_2 at 37°C (normoxic conditions) for 2 hours as reoxygenation.

Cell viability assay

After OGD/R, cell viability was assessed using the Cell Counting Kit-8 (CCK8; targetmol, USA). H9c2 cells were seeded in 12-well plates (1×10^5 cells/ml) and subjected to OGD/R, and then the cells were incubated with 10% CCK-8 solution at 37°C for 1 hour under normoxia, and the absorbance (by TECAN Sunrise ELISA Reader detects ODs) at 450 nm to calculate cell viability. Cell viability is expressed as a percentage of the absorbance of control-treated cells exposed to normoxic conditions.

Detection of ROS amounts and inflammatory cytokines

The effect of *D. salina* on the cellular level of reactive oxygen species (ROS) was analyzed using the Cellular ROS/Superoxide Detection Assay KIT (Abcam, Cambridge, UK) following the manufactures instructions. The levels of TNF-α and IL-1β were determined using enzyme-linked immunosorbent assay (ELISA) kits (Abcam, Cambridge, UK) according to the manufacturer's protocols.

Supplementary Figure

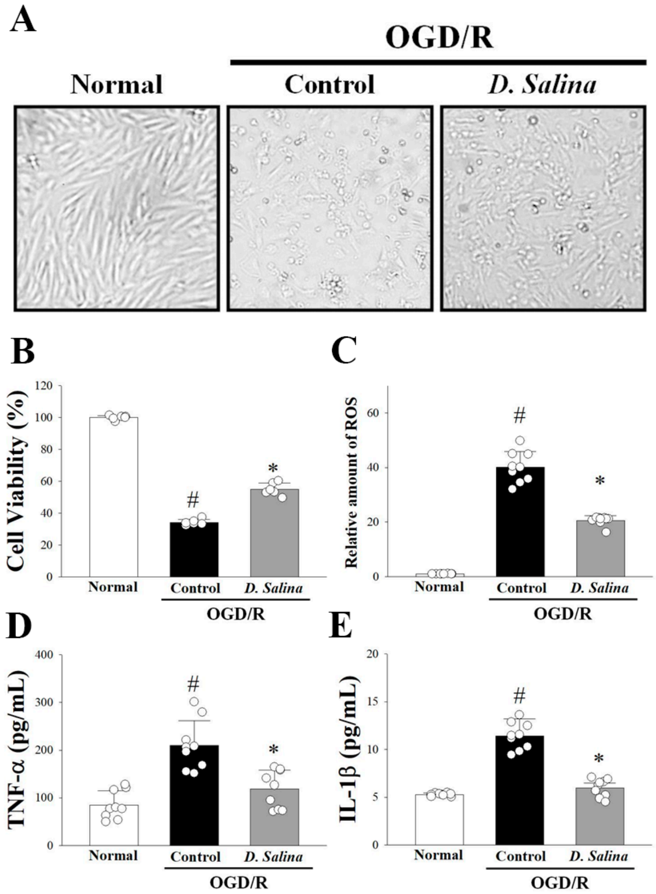

Figure S1. The protective effect of *D. Salina* in H9c2 cells subjected to OGD/R. (A) Morphology alterations in H9c2 cells treated with or without *D. Salina* (10μM) after OGD/R injury. (B) Quantitative the effects of *D. Salina* against OGD/R-induced decrease of cell viability by CCK-8 assay. (C) Quantitative the effects of *D. Salina* on the relative amount of ROS in H9c2 cells after OGD/R injury by ROS/superoxide detection assay kit and normalized with the mean value from the normal group. Evaluation the effects of *D. Salina* on the inflammatory cytokines (D) TNF-α and (E) IL-1β in H9c2 cells after OGD/R injury by enzyme-linked immunosorbent assay kit. Data are expressed as means ± SD (n = 9). #p < 0.05 compared with the normal group;*p < 0.05 compared with the OGD/R-control group.
